# Monocyte-derived factors including PLA2G7 induced by macrophage-nasopharyngeal carcinoma cell interaction promote tumor cell invasiveness

**DOI:** 10.18632/oncotarget.10980

**Published:** 2016-08-01

**Authors:** Heng Boon Low, Chin Wen Png, Chunwei Li, De Yun Wang, Soon Boon Justin Wong, Yongliang Zhang

**Affiliations:** ^1^ Department of Microbiology and Immunology, Yong Loo Lin School of Medicine, National University of Singapore, Singapore 117545, Singapore; ^2^ Immunology Programme, the Life Science Institute, National University of Singapore, Singapore 117597, Singapore; ^3^ Department of Otorhinolaryngology, the First Affiliated Hospital of Sun Yat-Sen University, Guangzhou 510080, China; ^4^ Department of Otolaryngology, Yong Loo Lin School of Medicine, National University of Singapore, Singapore 117597, Singapore; ^5^ Department of Pathology, National University Hospital, Singapore 119074, Singapore

**Keywords:** nasopharyngeal cancer, tumor associated macrophage, inflammation

## Abstract

The non-keratinizing undifferentiated subtype of nasopharyngeal carcinoma (NPC) is a malignancy characterized by an intimate relationship between neoplastic cells and a non-neoplastic lymphoid component. Tumor-associated macrophages (TAMs) foster tumor progression through production of soluble mediators that support proliferation, angiogenesis, survival and invasion of malignant cells. However, the role of macrophages in the progression of NPC remains poorly understood. This study aims to investigate the functional and phenotypic changes that occur to macrophages in macrophage-NPC cell co-culture systems, and how these changes influence tumor cells. We found that monocytes, including THP-1 cells and primary human monocytes, co-cultured with C666-1 NPC cells upregulate expression of pro-inflammatory cytokines at the early stages, followed by the induction of metastasis-related genes and interferon-stimulated genes at the later stage of coculture, indicating that TAMs are “educated” by NPC cells for cancer progression. Importantly, the induction of these factors from the TAMs was also found to enhance the migratory capabilities of the NPC cells. We have also identified one of these macrophage-derived factor, phospholipase A2 Group 7 (PLA2G7), to be important in regulating tumor cell migration and a novel tumor-promoting factor in NPC. Further studies to characterize the role of PLA2G7 in tumor metastasis may help determine its potential as a therapeutic target in NPC.

## INTRODUCTION

Leukocyte infiltration into tumor tissues is a hallmark of most forms of malignancies [1]. These cells, together with fibroblasts and vascular endothelial cells form the tumor microenvironment [2]. Macrophages infiltrate into malignant tumor tissues in high numbers. These tumor-associated macrophages (TAMs) have emerged as central regulators of both tumor onset and progression [3]. Macrophages are loosely classified into M1 (classically activated) and M2 (alternatively activated) macrophages according to their polarization state [4, 5]. M1 macrophages are activated by microbial products and are characterized by the release of inflammatory cytokines, reactive nitrogen intermediates (RNI), reactive oxygen intermediates (ROI), and microbicidal/tumoricidal activity. In contrast, M2 macrophages are polarized by anti-inflammatory cytokines (such as IL-4, IL-13, and IL-10), apoptotic cells, and immune complexes to display an immunosuppressive phenotype and are characterized by enhanced release of anti-inflammatory cytokines, scavenging potential and the ability to promote angiogenesis, tissue remodeling, and repair [4, 6, 7]. It is believed that the majority of TAMs are derived from blood monocytes, recruited to tumors by chemokines such as MCP-1/CCL2 [1]. The recruited monocytes then undergo a ‘reprogramming’ process, which is dependent on signals from the tumor microenvironment. Although tumors from diverse tissues are infiltrated by phenotypically and functionally distinct TAM populations [8], the infiltration of TAMs or the enrichment of TAM-associated gene signatures correlates with poor prognosis and disease outcome in most (but not all) human tumor types [1], suggesting that TAMs have pro-tumor functions.

Although early studies indicate TAMs to have a M2-like phenotype with tumor promoting functions, there is growing evidence that the phenotype of the macrophages depends on the stage of tumor development. Macrophages displaying a tumorigenic, M1-like phenotype are often found at sites of chronic inflammation, whereas the tumor-promoting, M2-like macrophages are found in established tumors [5, 6, 9]. In addition, mixed phenotypes of TAMs that co-express proinflammatory genes such as TNF and IL-1 together with known tumor-promoting genes including VEGFA and MMP9 have been observed in various tumor models including renal cell carcinoma and mammary adenocarcinoma [10]. The complexity of TAMs poses substantial challenge for targeting these cells for anti-cancer therapy. Further investigation on the detailed mechanisms of the pro-tumor activity of TAMs is necessary for the advancement of TAM-directed anti-cancer therapy [2].

Nasopharyngeal carcinoma (NPC) is a malignancy arising from epithelial cells that line the nasopharynx, the uppermost region of the pharynx continuous with the nasal cavities [11]. Although the incidence of NPC in western countries is rare, NPC is particularly common in Southern China, Southeast Asia and North Africa [11–13]. Studies on geographic and racial variations in NPC incidence suggest a multifactorial etiology. In endemic populations, risk appears to be due to an interaction of several factors: Epstein-Barr virus (EBV) infection, genetic predisposition, and environmental factors such as the high intake of preserved foods and smoking [14–18]. According to the World Health Organization (WHO), NPC can be histologically classified into 3 subtypes; keratinizing squamous cell carcinomas (Type I), non-keratinizing squamous cell carcinoma (Type II), and undifferentiated carcinoma (Type III) [19]. The undifferentiated form of NPC is most common and has the strongest association with EBV [20].

NPC represents a unique tumor microenvironment, where epithelial tumor cells flourish among abundant infiltrating immune cells. Previous studies have reported intense leukocyte infiltration comprising of mainly macrophages and T cells in NPC [21, 22]. The T lymphocytes present in tumor tissues have been reported to have impaired cytotoxic function [23] and the antitumor effects of T cells may be inhibited by immunosuppressive factors produced by TAMs and immature myeloid cells (such as myeloid-derived suppressor cells) [7, 24]. Additionally, recruitment of T regulatory cells (Treg) to the tumor site can be also be suppressed by TAM [22, 25]. Though it is well-accepted that TAMs play significant roles in promoting tumorigenesis, the specific involvement of macrophages in NPC tumor tissue has not been well characterized.

In this study, we hypothesize that interactions between macrophages and NPC cells promote the establishment of a pro-inflammatory microenvironment that subsequently induces the upregulation of tumorigenic factors in both macrophages and NPC to promote the development of cancer. Indeed, our study shows that macrophage-NPC cell interaction resulted in upregulation of genes involved in inflammation as well as NPC metastasis. Novel targets such as phospholipase A2 group VII (PLA2G7) that may contribute to tumor progression were also identified from our screen. The valuable information about macrophage-NPC interaction from our study will provide a basis for further in-depth characterization and development of potential therapies for NPC.

## RESULTS

### Co-culture of THP-1 and C666-1 cells induced proinflammatory cytokine gene expression with differential expression kinetics

**Figure 1:**
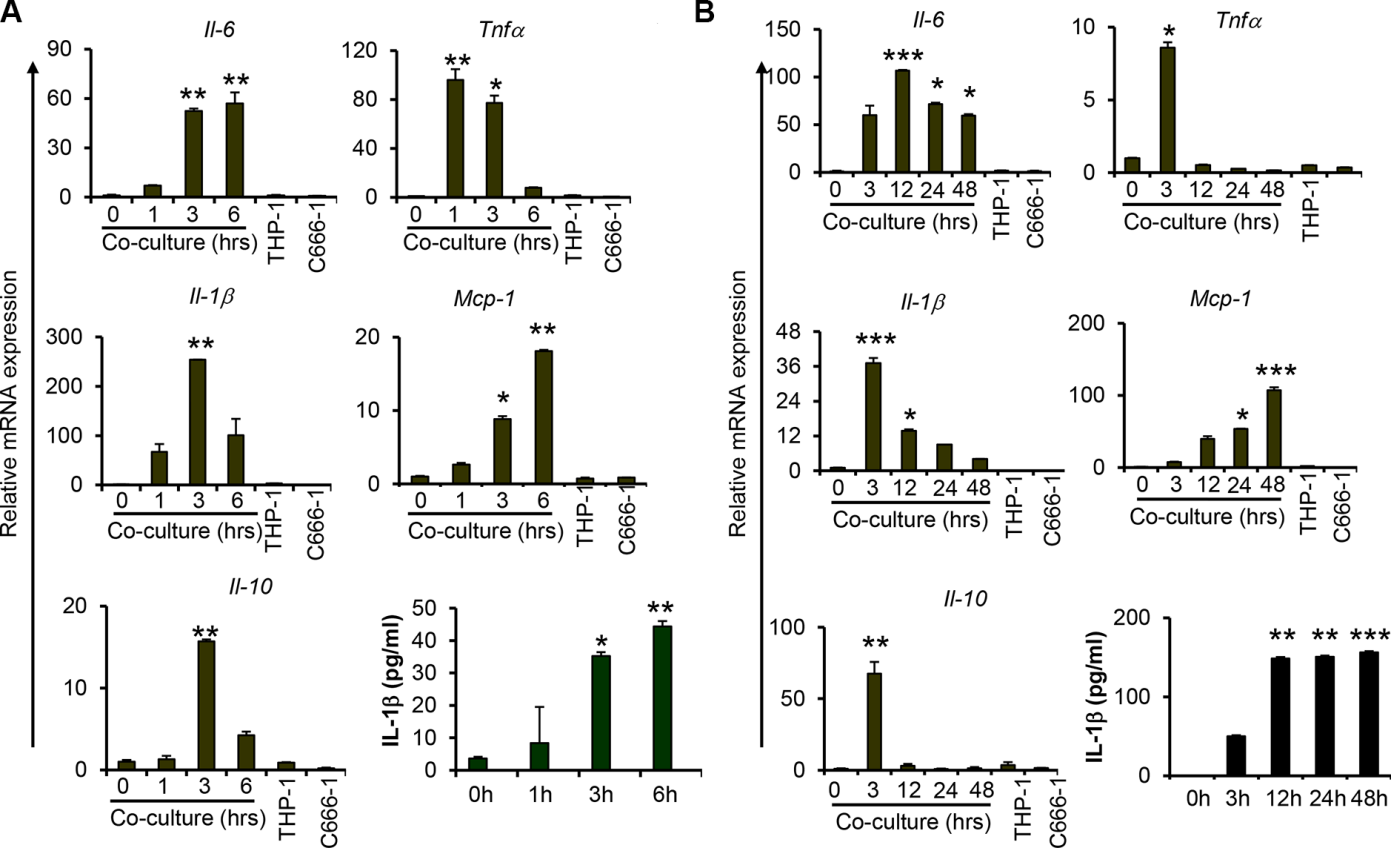
Co-culture of macrophages with nasopharyngeal carcinoma cells increases proinflammatory cytokine expression Relative gene expression levels of proinflammatory cytokines, *Il6*, *Tnfa, Il-1b*; chemokine *Mcp-1;* anti-inflammatory cytokine *Il-10* and protein levels of IL-1b were measured from undifferentiated THP1 cells co-cultured with C666-1 at both (**A**) early and (**B**) late/extended time points. Relative gene expression levels were determined by quantitative real-time PCR (qPCR). Protein expression of IL-1b was determined by ELISA. The images shown are a representative set from 2-3 experiments *P < 0.05, **P < 0.01, ***P < 0.001 (mean ± S.D, *n* = 3).

To examine the interplay between macrophages and NPC cells, undifferentiated THP-1 monocytic cells were co-cultured with the EBV-positive NPC cell line, C666-1, in a direct contact system to examine the expression of proinflammatory cytokines including IL-6, TNFα and IL-1β, and chemokine MCP-1, as well as anti-inflammatory cytokine IL-10. We observed increased mRNA expression of these cytokines and chemokine at 1 hour (hr) post co-culture of THP-1 and C666-1 cells (Figure 1A). The highest expression of TNFα was detected at 1hr, whereas the expression of IL-1β and IL-10 peaked at 3 hrs post co-culture. Similar results were obtained from co-culture of PMA-differentiated THP-1 macrophages with C666-1 (Figure S1A, S1B). In addition, we observed sustained expression of IL-6 throughout the duration of co-culture, with the highest expression observed at 12 hrs of co-culture (Figure 1B). The expression of TNFα, on the other hand, was reduced to a basal level after 12 hrs of co-culture. Interestingly, the expression of MCP-1 was progressively increased, with the highest expression detected at 48 hrs of co-culture. mRNA expression of IL-1β was found to be greatly reduced after 12 hrs, whereas its protein expression was induced as early as 3 hrs and sustained throughout the entire period of co-culture (Figure 1A, 1B). Similar to TNFα, the induction of IL-10 expression appears to be transient. Its expression peaked at 3 hrs and returned to a basal level at 12 hrs of co-culture (Figure 1B). In addition, we examined the expression of TGFβ, another immunosuppressive cytokine frequently identified in tumor microenvironment, and found that its expression was not induced by THP-1-NPC cell interaction (Figure S2). Together, these results demonstrate that although the expression of these proinflammatory and anti-inflammatory mediators are induced at the early stage of macrophage-NPC interaction, they are differentially regulated as the contact co-culture progresses. Interestingly, the expression of Arginase I (*Arg1*), which is considered a marker of macrophage alternative activation (M2 activation) [26], was moderately increased at 3 and 12 hrs, and returned to basal level after 24 hrs of co-culture (Figure S2).

### Increased expression of metastasis-related and interferon-stimulated genes during macrophage-NPC cell interaction

**Figure 2:**
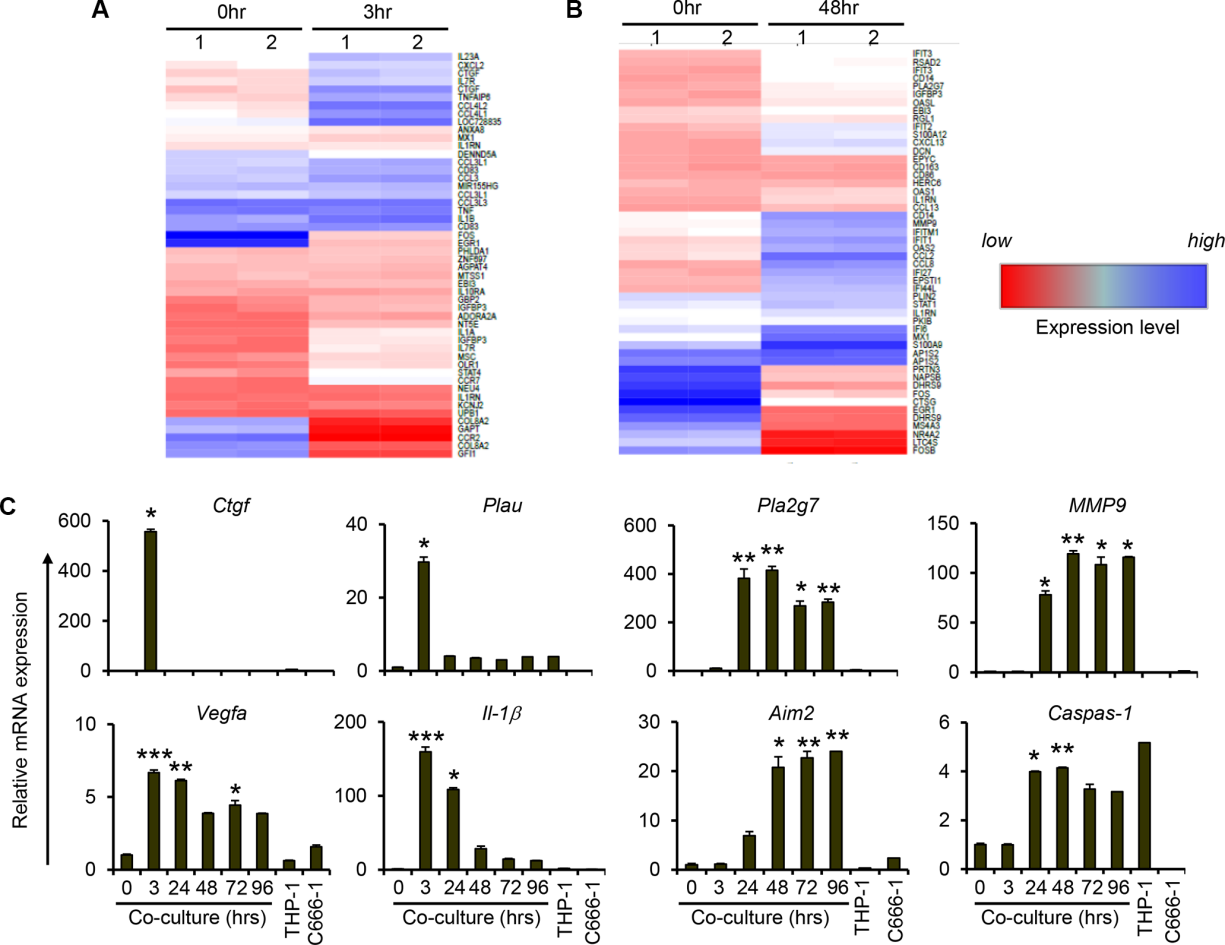
Increased expression of genes related to cancer initiation, metastasis and inflammasome during prolonged interaction between THP-1 and NPCs Undifferentiated THP-1 cells were co-cultured with C666-1 cells for (**A**) 3 hr or (**B**) 48 hr. Changes of gene expression compared to 0 hr of co-culture is depicted. “Blue” and “red” colors represent high and low levels of gene expression, respectively. (**C**) Expression of genes related to cancer initiation and metastasis, and inflammasome-related genes (AIM2 and CASP1) from co-culture of THP-1 and C666-1 cells over 4 days was assessed by qPCR. The qPCR data shown is a representative set from 2 experiments (mean ± S.D, *n* = 3) *P < 0.05, **P < 0.01, ***P < 0.001 (mean ± S.D, *n* = 3).

To determine the global changes in gene expression during short-term and long-term co-culture of macrophages and NPC cells, we performed microarray analysis on 3 hr- and 48 hr-co-culture samples. Compared to controls (0h), there was increased expression of various proinflammatory cytokines and chemokines after 3 hrs of co-culture (Figure 2A). In agreement with our previous results (Figure 1), increased expression of inflammatory cytokines including TNFα and IL-1β was observed from the microarray data. In addition, increased expression of other cancer promoting genes such as connective tissue growth factor (CTGF), urokinase-type plasminogen activator (PLAU) and CD44 was observed at 3 hrs of co-culture (Figure 2A and Table S1) Interestingly, we observed increased expression of other chemokine genes including CCL8 and CXCL13, metastasis-related genes such as MMP9 and PLA2G7, and various interferon-stimulated genes such as IFITs, IFIs and OAS after 48 hrs, but not 3 hrs, of co-culture (Figure 2B). Thus the microarray data also provides evidence of a change in phenotype of the cells in the tumour microenvironment as cell interaction progresses.

### Sustained metastasis-related gene expression during macrophage-NPC cell interaction

Co-culture of THP-1/C666-1 cells was extended to 4 days to validate the microarray results and to assess further changes in gene expression profile during monocyte/macrophage-NPC cell interaction. Consistent with the microarray data, expression of CTGF and PLAU was upregulated at 3 hrs of co-culture and subsequently returned to the basal levels (Figure 2C). On other hand, sustained expression of metastasis-related genes, including PLA2G7, MMP9 and VEGFA, was observed throughout the 4 day duration of co-culture. In line with increased expression of IL-1β, we observed increased expression of inflammasome gene AIM2 and its target gene caspase-1 (Figure 2C). In addition, the expression of proinflammatory mediators, including MCP-1 and IL-6, increased steadily up to day 2 of co-culture (Figure S3). Subsequently, the expression levels of these genes decreased but still showed significantly greater expression relative to the controls (0 hr of co-culture). The expression of TNFα and IL-10, on the other hand, showed only transient induction up to 3 hrs of co-culture (Figure S3).

Overall, these results indicate that prolonged interaction between monocytes/macrophages and NPC cells results in phenotypic changes in macrophages, NPC cells or both, including upregulation of genes that may favor the progression and aggressiveness of NPC cells.

### Sustained expression of inflammatory and metastasis-related gene expression requires direct interaction between macrophages and NPC cells

**Figure 3:**
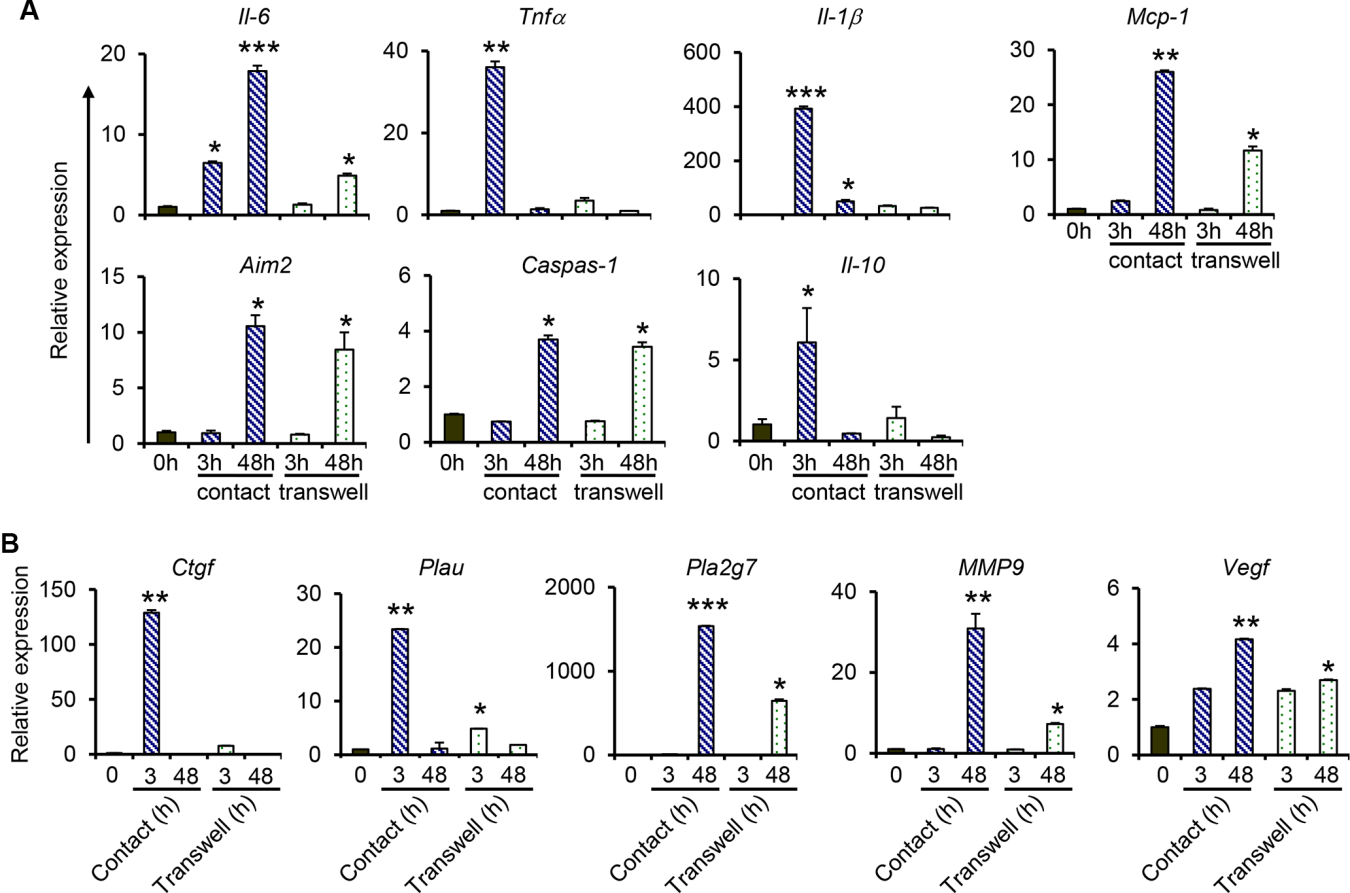
Comparison of gene expression between contact and non-contact co-cultures of THP-1 and NPC cells For contact co-culture, THP-1 cell were added directly into culture wells containing C666-1 cells. For non-contact (transwell) co-culture, 0.2 × 10^6^ C666-1 cells were cultured in the lower well of a 6-well plate and 0.2 × 10^6^ THP-1 cells were seeded in the upper inserts. The co-cultures were maintained for 3 hrs or 48 hrs. Relative expression of genes related to (**A**) inflammation, and (**B**) cancer-initiation and metastasis, was determined by qPCR. The data shown is representative of set from 2 experiments *P < 0.05, **P < 0.01, ***P < 0.001 (mean ± S.D, *n* = 3).

To investigate if direct contact between monocytes/macrophages and NPC cells was required for the expression of proinflammatory and metastasis-related genes, THP-1 cells were co-cultured with C666-1 cells in tissue culture plates with Transwell inserts (non-contact co-culture), and gene expression was examined after 3 hrs or 48 hrs. The results show that non-contact co-culture of THP-1 cells with NPC cells induces the expression of IL-6, TNFα, IL-1β and MCP-1 (Figure 3A), however at significantly lower levels compared to that induced by contact co-culture. For instance, the induction of IL-6 expression (about 5-fold) is only observed at 48 hrs, but not at 3 hrs, of co-culture in the non-contact co-culture system, whereas a 6.5-fold and 18-fold induction was observed at 3 hrs and 48 hrs, respectively in the contact co-culture (Figure 3A). Similarly, a 36-fold induction of TNFα was observed at 3 hrs in the contact co-culture, whereas that in the non-contact co-culture was only 3.5-fold. Similar patterns were observed in the expression of MCP-1 (Figure 3A). IL-10 expression was only induced at 3 hr in the contact co-culture, but not in the non-contact co-culture. While contact co-culture greatly induced the expression of IL-1β at 3 hrs (392-fold), decreasing to about 50-fold at 48 hrs, the non-contact co-culture only led to a 32.6-fold and 26-fold induction at 3 hrs and 48 hrs of co-culture, respectively. Interestingly, the expression of AIM2 and Caspase-1 was similar in contact and non-contact co-culture. Both genes were only induced at 48 hrs in the two systems and there was little to no difference in their expression levels in both co-culture systems (Figure 3A). Together, these results demonstrate that soluble factors from macrophages and/or NPC cells are able to induce low levels of proinflammatory gene expression, and direct interaction between macrophages and NPC cells is more potent in inducing expression of these genes.

Similarly, direct interaction between macrophages and NPC cells is required for the induction of high-level expression of metastasis-related genes. Consistent with data depicted in Figure 2C, the expression of both CTGF and PLAU was only observed at 3 hrs in the co-culture systems (Figure 3B). A 128.9-fold vs 7.7-fold induction of CTGF expression in the contact vs non-contact co-culture was observed. For PLAU expression, a 23-fold induction was observed in the contact co-culture, which was higher than the 4.8-fold induction in the non-contact co-culture. Expression of PLA2G7 and MMP9 was only induced at 48 hrs in both co-culture systems and significantly higher induction of both genes was detected in the contact co-culture compared to the non-contact co-culture (Figure 3B) Similar levels of expression of VEGFA were observed at 3 hrs in both co-culture systems. Its expression was further increased at 48 hrs in the contact co-culture, which was not observed in the non-contact co-culture.

### Primary human monocytes co-cultured with NPC cells induce the expression of proinflammatory and metastasis-related genes

**Figure 4:**
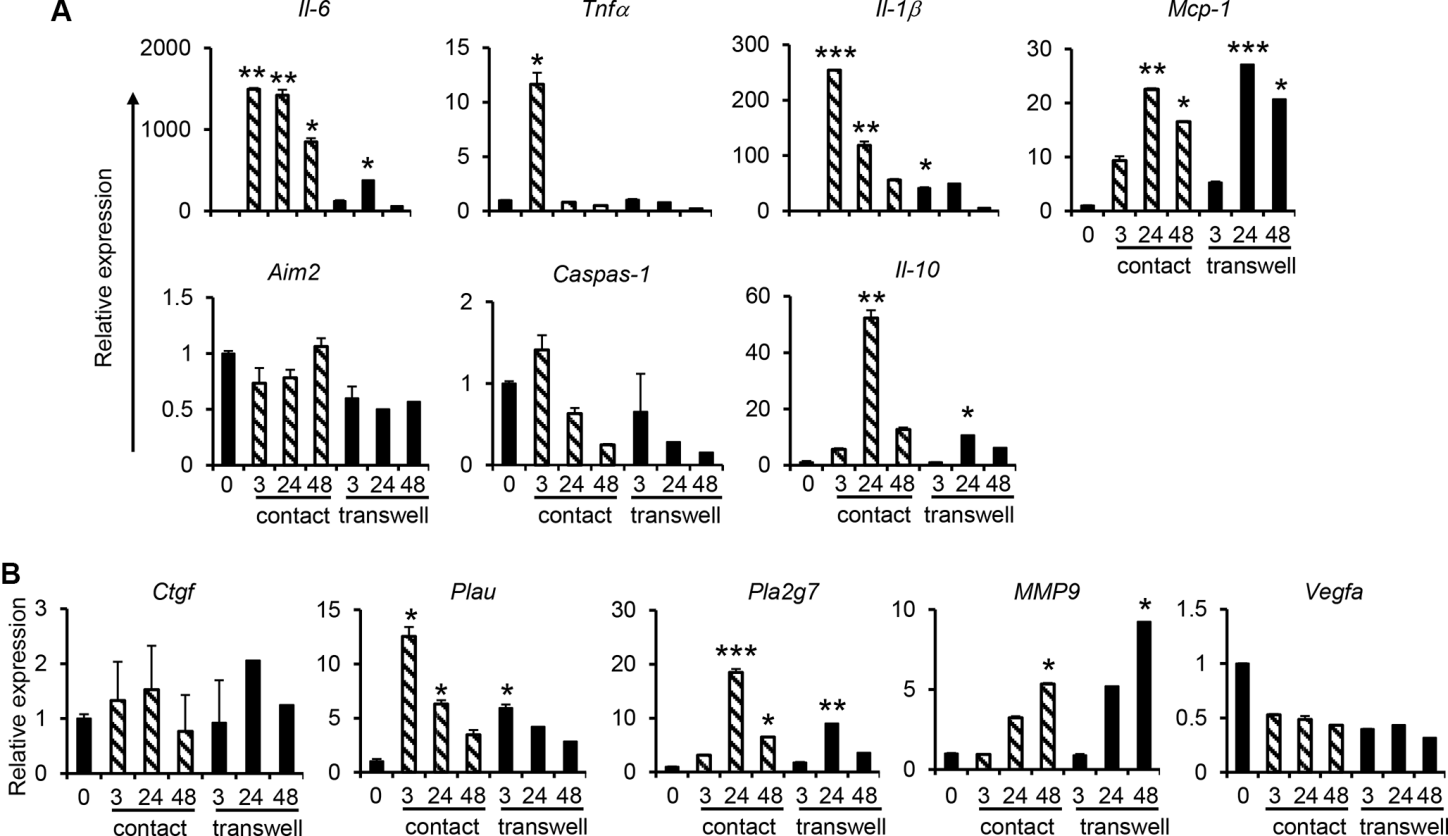
Increased expression of pro-inflammatory and cancer-promoting genes during co-culture of primary human monocytes with NPC cells Human monocytes derived from PBMCs of healthy individuals were co-cultured with C666-1 cells in direct contact and transwell systems. Expression of (**A**) inflammation-related genes, or (**B**) cancer-promoting genes, was assessed by qPCR. The results shown are representative of a set of 2 experiments (mean ± S.D, *n* = 3) *P < 0.05, **P < 0.01, ***P < 0.001 (mean ± S.D, *n* = 3).

Monocytes isolated from human peripheral blood mononuclear cells (PBMCs) by magnetic cell sorting using CD14 microbeads were co-cultured with C666-1 cells in both contact and non-contact co-culture systems. Compared with the THP-1/C666-1 co-culture, similar gene expression profiles of IL-6, TNFα, IL-1β and MCP-1 as well as metastasis-related genes including PLA2G7 and MMP9 in primary monocytes/C666-1 co-culture were observed (Figure 3 and Figure 4). For instance, sustained expression of IL-6, MCP-1, PLA2G7 and MMP9 was observed, whereas expression of TNFα was transient when either primary monocytes or THP-1 cells were co-cultured with C666-1 (Figure 1, Figure 3 and Figure 4A). Likewise, expression of proinflammatory, anti-inflammatory and metastasis-related genes in primary monocytes co-cultured with C666-1 in the non-contact co-culture system was much lower compared to the contact co-culture system (Figure 4A, 4B). However, unlike the THP-1/C666-1 co-culture, expression of AIM2 and CTGF in the primary monocyte/C666-1 co-culture did not change across the time points examined in both contact and non-contact co-culture systems (Figure 4A, 4B). In addition, reduced expression of VEGFA was observed in monocyte/C666-1 co-culture compared to that in THP-1/C666-1 co-culture (Figure 4B).

### Macrophages are the main contributors to the induction of proinflammatory and metastasis-related genes

Next we investigated the relative contributions of macrophages and NPC cells to the induction of cytokines and metastasis-related genes during macrophage-NPC cell interaction in both contact and non-contact co-culture systems. Therefore, we co-cultured THP-1 cells with C666-1 in the non-contact co-culture system to examine gene expression in THP-1 and C666-1 cells separately. In THP-1 cells, early (up till 6 hrs) upregulation and subsequent downregulation of proinflammatory cytokine genes including IL-6, TNFα, IL-1β and MCP-1 was observed (Figure S4A). In addition, increased and sustained expression of the metastasis-related genes such as PLA2G7, PLAU and MMP9 was observed (6 hrs and later) (Figure S4B). In contrast, induction of IL-6, TNFα, and IL-1β genes was not observed in C666-1 cells (Figure S4A), whereas the induction of PLAU and MMP9 was only observed at 72 hrs and 96 hrs (Figure S4B). Of note, the expression of PLA2G7 was not detected in C666-1 cells throughout the co-culture (Figure S4B). The results indicate that monocyte/macrophage-NPC cell interaction leads to induction of proinflammatory cytokines and metastasis-related genes mainly in THP-1 cells.

**Figure 5:**
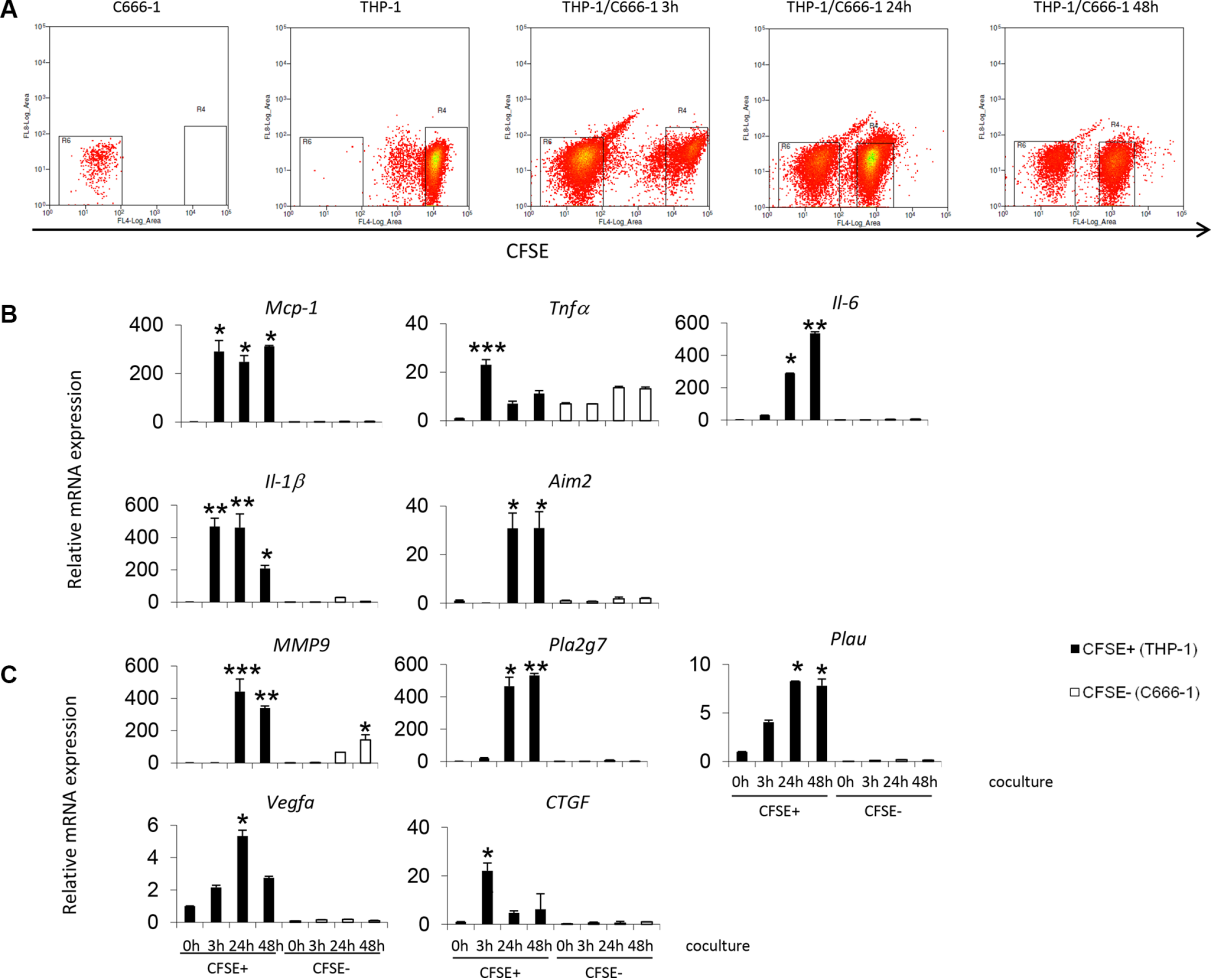
Gene expression by THP-1 cells or NPC cells during contact co-culture (**A**) Undifferentiated THP-1 cells were stained with CFSE prior to co-culture with C666-1 cells. After co-culture, the cells were sorted into CFSE positive (THP-1) and CFSE negative (C666-1) fractions. Expression levels of (**B**) inflammatory or (**C**) cancer-promoting genes by THP-1 cells or C666-1 cells were determined by qPCR. The qPCR data shown is a representative set of 2 experiments (mean ± S.D, *n* = 3) *P < 0.05, **P < 0.01, ***P < 0.001 (mean ± S.D, *n* = 3).

To further investigate the gene expression profile of the THP-1 and C666-1 cells individually after contact co-culture, THP-1 cells were stained with Cell Trace Carboxyfluorescein Succinimidyl Ester (CFSE) and co-cultured with unstained C666-1 cells. The CFSE+ stained cells (THP-1) were purified by flow cytometry cell sorting (Figure 5A) and the gene expression profiles of THP1 and C666-1 were assessed individually. The results confirm that the induction of cytokine and cancer-related genes upon co-culture was observed mainly in the THP-1 cells (CFSE positive fraction) (Figure 5B, 5C). In THP-1 cells, early (up till 6 hrs) upregulation and subsequent downregulation of proinflammatory cytokine genes including TNFα and IL-1β was observed (Figure 5A). The increased expression of cytokine genes such as MCP-1 and IL-6, and metastasis-related genes such as PLA2G7, PLAU and MMP9 was also observed and sustained through the duration of co-culture (6 hrs and later) (Figure 5B). Interestingly, increased expression of MMP-9 was also observed in the C666-1 cells at 24 hrs after co-culture and its expression continuously increased at 48 hrs, which suggests that these factors may be expressed by both cell types upon interaction to promote tumor aggressiveness (Figure 5B, 5C).

### Soluble factors from NPC cells induce proinflammatory and metastasis related gene expression by macrophages

It is known that macrophages are “educated” by tumor cells in the tumor microenvironment to promote cancer progression and metastasis [3]. To examine the role of soluble factors derived from NPC cells in the induction of proinflammatory and metastasis-related genes in monocytes/macrophages, we cultured THP-1 cells with conditioned medium from C666-1 culture to examine the expression of these genes. As shown in Figure S5A and S5B, NPC-conditioned medium was able to induce the expression of both proinflammatory and metastasis related genes in macrophages. However, compared with our co-culture results (Figures 1, 5 and S4), induction of IL-6 expression was delayed. Increased IL-1β expression was first detected at 3 hrs and maintained at lower level from 12 hrs onwards, in contrast to the sustained high level of IL-1β expression in THP-1/C666-1 co-culture. Similarly, induction of TNFα and PLA2G7 expression was lower in THP-1 cells cultured with C666-1 conditioned medium (Figure S5A and S5B). These results demonstrate that NPC-derived soluble factors are able to induce both proinflammatory and metastasis related genes in macrophages that might influence cancer progression; albeit at lower levels compared to direct interaction between macrophages and NPC cells.

### Macrophage-derived factors together with PLA2G7 enhance NPC cell migration

The increased expression of the metastasis-related genes prompted the investigation of the effect of the soluble factors produced by macrophages on cell migration. The migration of the C666-1 cells was assessed by the ability of the cells to migrate across a transwell insert with 8μm pores. Conditioned media from THP-1 cells, and co-cultures of THP-1 and C666-1 cells were used to represent the soluble factors produced by the THP-1 cells.

**Figure 6:**
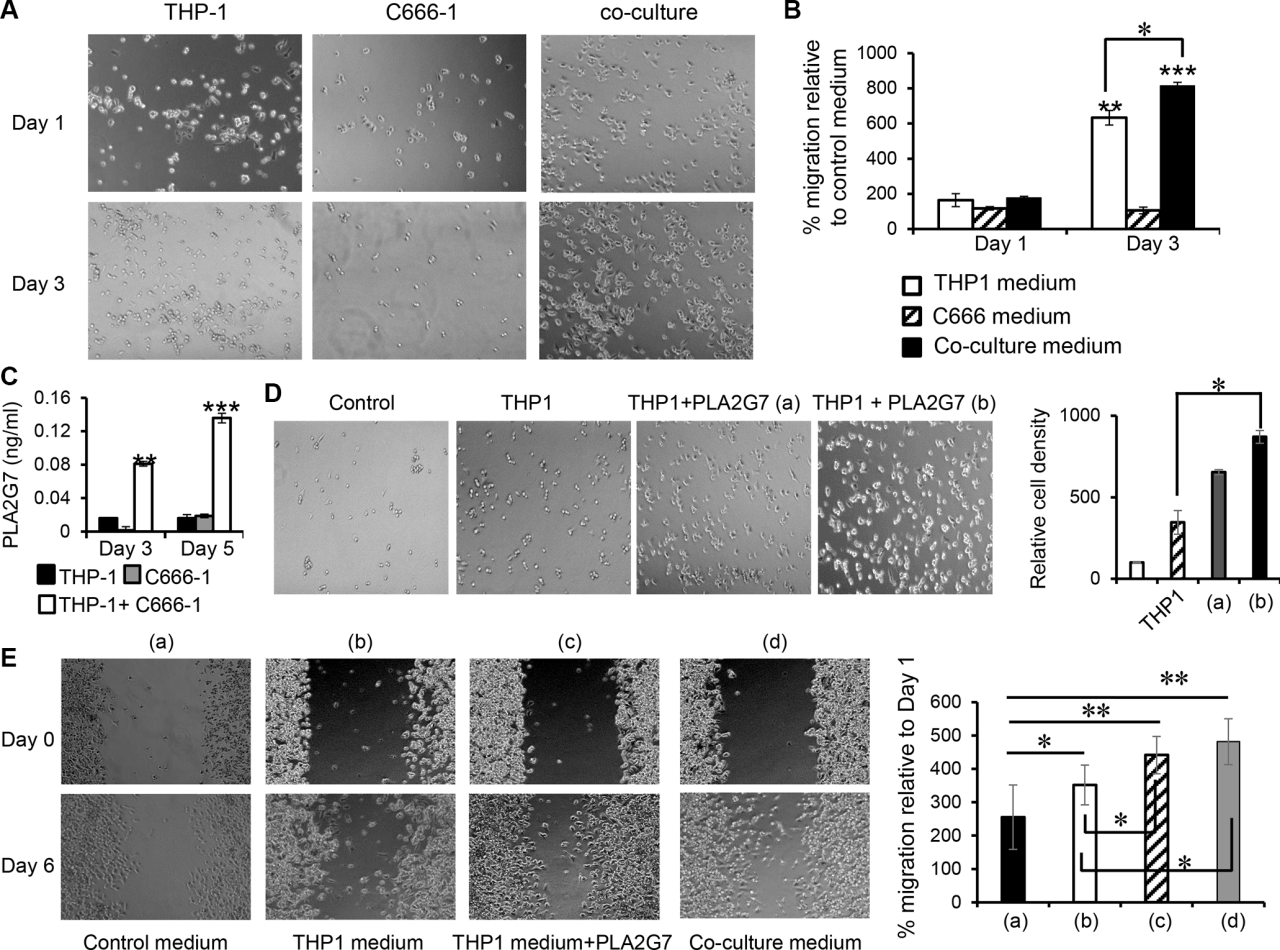
Culture supernatant from THP-1/C666-1 co-culture promotes NPC cell migration (**A**) C666-1 cells were seeded into a transwell (upper insert) with 8mm pores. Complete medium, THP-1 conditioned medium, C666-1 conditioned medium or conditioned medium from THP-1/C666-1 co-culture was added to the lower wells of the plate and migration of the C666-1 cells across the transwell was assessed. (**B**) Representative micrographs and the bar chart show migrated C666-1 cells and cell counts in the lower chamber (migrated cells) relative to the control (complete RPMI medium only) respectively. The images shown are representative of a set of 6 images (3 frames from 2 experiments) and data shown are representative of 2 experiments. (**C**) Protein expression of PLAG7 was determined by ELISA from culture media of THP-1 cells, C666-1 cells and THP-1/C666-1 co-cultures. The data shown is representative of 3 experiments. (**D**) THP-1 conditioned medium containing 40 pg/mL (a) or 80 pg/mL (b) of recombinant PLA2G7 were added to the lower wells of the plate and migration of the C666-1 cells across the transwell was assessed. Representative micrographs and the bar chart show migrated C666-1 cells and cell counts in the lower chamber (migrated cells) relative to the control (complete RPMI medium only) respectively. Images shown are representative of 2 experiments. (**E**) Wound healing of C666-1 monolayer in response to control medium (a), THP-1 conditioned medium (b), THP-1 conditioned medium plus 80 pg/ml of recombinant PLA2G7 (c), or THP-1/C666-1 co-culture conditioned medium (d) was performed. Images shown are representative of 2 experiments. *P < 0.05, **P < 0.01, ***P < 0.001 (mean ± S.D, *n* = 3).

The number of migrated C666-1 cells increased by 6- and 8- fold (*P* < 0.01) in conditioned medium from THP-1 cells cultured alone for 3 days, and conditioned medium from 3 days-co-culture of THP-1 and C666-1 respectively compared to the control medium (Figure 6A and 6B, Complete RPMI vs Day 3 conditioned media from THP-1 cells: 100% vs 632%, *p* < 0.01; Complete RPMI vs Day 3 conditioned media from co-culture of THP-1 and C666-1 cells: 100% vs 814%, *p* < 0.01). In contrast, there was no significant difference in cell migration for the conditioned medium from THP-1 cells cultured alone for 1 day, and conditioned medium from 1 day co-culture of THP-1 and C666-1 respectively compared to the control medium (Figure 6A and 6B). Conditioned media from both Day 1 and Day 3 cultures of C666-1 did not significantly affect the migration of the NPC cells. These results indicate that soluble factors produced by THP-1 are able to increase the migration capabilities of NPC cells. The results also show that the increased expression of factors resulting from the interaction THP-1 and C666-1 cells was able to further enhance the migration capabilities of NPC cells.

We observed inducible, prolonged expression of PLA2G7, a secreted enzyme that is associated with pathogenesis of prostate and colorectal cancers [27, 28], from THP-1 cells after co-culture with C666-1 cells or its conditioned medium. However, its function in NPC is unclear. To assess the function of this molecule in NPC, firstly, we assessed the production of PLA2G7 protein and found that co-culture of THP-1 and C666-1 cells led to a 1.8- and 3.4-fold increase in PLA2G7 protein expression compared to THP-1 or C666-1 cells cultured alone respectively after 3 days and a 4.64 and 3.9-fold increase in PLA2G7 protein expression compared to THP-1 or C666-1 cells cultured alone respectively after 5 days (Figure 6C), demonstrating that monocyte/macrophage-NPC cell interaction leads to increased expression of PLA2G7.

Next, we tested the ability of this enzyme to regulate the migration of NPC cells. Interestingly, THP-1 conditioned media alone led to 3.4-fold greater C666-1 migration compared to control media (Control media vs. THP-1 conditioned media: 100% vs 346%) while THP-1 conditioned media supplemented with 40 pg/ml or 80 pg/ml of PLA2G7 was able to induce 6.5-fold or 8.7-fold greater migration of C666-1 cells compared to control media respectively (Control media vs. THP-1 conditioned media supplemented with 40 pg/ml or 80 pg/ml of PLA2G7: 100% vs 654%, or 100% vs 870%), demonstrating that PLA2G7 was able to further enhance NPC cell migration (Figure 6D).

We then performed wound healing assay to substantiate the findings above. We observed that THP-1conditioned media was able to increase wound healing of C666-1 monolayer compared to control media (Figure 6E). THP-1 conditioned media supplemented with 80 pg/ml of PLA2G7 significantly enhanced C666-1 wound healing compared to that of THP-1 conditioned media alone. Similarly, conditioned media from THP-1/C666-1 co-culture significantly increased wound healing of C666-1 monolayer (Figure 6E).

**Figure 7:**
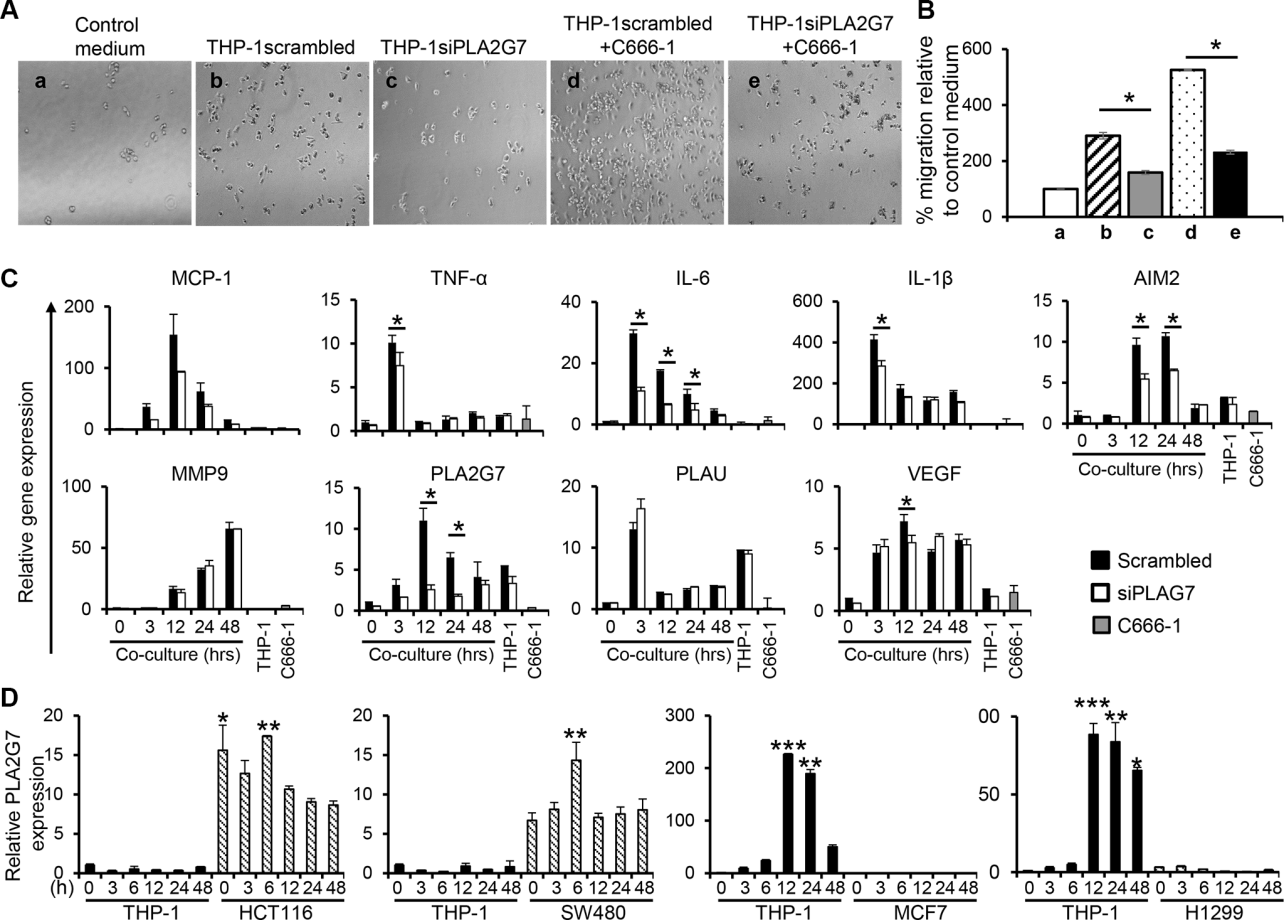
PLA2G7 silencing leads to reduction in cell migration and reduced induction of inflammatory genes *PLA2G7* gene silencing was carried out on THP-1 cells before co-culture with C666-1 cells. Conditioned media from control vector (THP-1scrambled), PLA2G7-silenced THP-1 cells (THP-1siPLA2G7), and THP-1scrambled or THP-1siPLA2G7 co-cultured with C666-1 cells were used to assess the C666-1 cell migration. (**A**) Representative micrographs and (**B**) the bar chart show migrated C666-1 cells and cell counts in the lower relative to the control (complete RPMI medium only). The changes in relative mRNA expression of (**C**) inflammatory and cancer-promoting genes in response to PLA2G7 siRNA transfection was determined by qPCR. (**D**) Undifferentiated THP-1 were co-cultured with colorectal cancer cell HCT116 and SW480, breast cancer cell MCF7 or non-small lung cancer cell H1299, respectively in a transwell system. Express of PLA2G7 was determined by qPCR. The results are representative of 2 experiments. *P < 0.05, **P < 0.01, ***P < 0.001 (mean ± S.D, *n* = 3).

To verify the function of PLA2G7 in promoting NPC cell migration, siRNA approach was employed to silence the expression of this gene in THP-1 cells. Conditioned media obtained from PLA2G7-silenced THP-1 cells (THP-1siPLA2G7) led to a 1.8-fold lower induction of C666-1 migration compared to conditioned media from THP-1 cells transfected with control scrambled siRNA (THP-1scrambled) (THP-1scrambled vs. THP-1siPLA2G7: 289% vs 158%) (Figure 7A and 7B). Furthermore, conditioned media from co-culture of THP-1siPLA2G7 and C666-1 cells showed 2.3-fold reduced induction of C666-1 migration compared with conditioned medium from THP-1scrambled co-cultured with C666-1 cells (THP-1scrambled+C666-1 vs. THP-1 siPLA2G7+C666-1: 525% vs 231%) (Figure 7A and 7B). In both cases, the co-culture conditioned media stimulated greater migration than the respective THP-1scrambled or THP-1siPLA2G7 monocultures. Co-culture of THP-1siPLA2G7 with C666-1 cells also showed reduced induction of PLA2G7 and inflammatory cytokine genes compared to co-culture of THP-1scrambled with C666-1 cells (Figure 7C). Together, these results demonstrate that macrophage-NPC cell interaction induces the expression of PLA2G7 by macrophages, which play an important role in NPC cell migration.

To examine the ability of other cancer cell types to induce PLA2G7 expression by monocytes/macrophages, we co-cultured THP-1 cells with two colorectal cancer (CRC) cell lines (HCT116 and SW480), one breast cancer cell line MCF7 and one non-small lung cancer cell line H1299 in a non-contact co-culture system. The expression of PLA2G7 in THP-1 cells or these cancer cells was examined. Interestingly, the two CRC cell lines, HCT116 and SW480, constitutively express PLA2G7 and did not induce THP-1 cells to express this gene (Figure 7D). On the other hand, both MCF7 and HCT116 cells, which do not expression PLA2G7 themselves, induced high expression of this gene in THP-1. These results suggest that PLA2G7 in tumor microenvironment could be from TAMs or tumor cells, which is dependent on the nature of the cancer cells.

## DISCUSSION

Macrophages constitute a major part of the leukocyte infiltrate in human cancers and the presence of these TAMs in the tumor microenvironment is associated with poor patient prognosis [29, 30]. TAMs may exert pro-tumoral functions by producing various cytokines, chemokines, growth factors and enzymes that promote tumor growth, angiogenesis, invasion, and/or metastasis [31]. Previous studies have provided evidence of intense leukocyte infiltration predominantly comprising macrophages and T cells in NPC tumor tissue [32]. These macrophages express various chemokines including MIP-1α and MCP-1 which could contribute to the recruitment of more leukocytes into the tumor [32]. However, the function of TAMs in NPC is not clear. In this study, we used both contact and non-contact co-cultures of THP-1 cells, or primary human monocytes, with C666-1, an undifferentiated NPC harboring the Epstein-Barr virus (EBV) genome, to investigate phenotypic changes of both macrophages and NPC cells during their interaction. We found that co-culture of THP-1 cells or human primary monocytes with C666-1 cells induced the expression of pro-inflammatory cytokine and chemokine genes including TNFα, IL-6, IL-1β and MCP-1, and anti-inflammatory cytokines such as IL-10 (Figures 1–5). The expression of some of these genes, including TNFα and IL-10, was reduced when the duration of co-culture was extended, whereas the expression of other genes, such as IL-6 and MCP-1, was sustained (Figure 1B, S3). Interestingly, several metastasis-related genes, including PLAU and PLA2G7, were found to be highly induced in monocytes/macrophages, but not in C666-1 cells (Figures 5 and S4), indicating that NPC cells are able to change the phenotype of macrophages to promote tumor metastasis.

Non-contact co-culture allows the study of the interaction between tumor cells and stromal cells through soluble factors. The expression of many of the genes such as TNFα, IL-6, IL-10, and MCP-1 exhibited similar kinetics between contact and non-contact co-cultures (Figure 3), but the levels of induction were much lower in non-contact co-culture, suggesting that soluble factors are sufficient for the induction of these genes and direct interaction between macrophages and NPC cells augments their expression.

Chronic inflammation is a potential contributor to carcinogenesis and is a feature of certain virus-associated cancers such as NPC [33]. We observed sustained expression of IL-6, IL-1β and MCP-1 in monocyte/macrophage-NPC co-culture systems and these proinflammatory mediators were found to be mainly produced by macrophages. IL-6 is known to promote cancer cell proliferation and survival [34]. In NPC, higher expression of IL-6 expression in tumor tissues was associated with poorer patient survival [35]. The sustained expression of IL-6 by monocytes/macrophages during their interaction with C666-1 cells suggests that NPC cells actively induce IL-6 production in macrophages, which may help to promote their own growth/survival.

IL-1β, a pleiotropic cytokine, is important for inflammation, immunity, hematopoiesis, and has tumor-promoting effects [36]. IL-1β expression has been reported in various cancers and increased local IL-1β levels usually correlate with tumor invasiveness and poor patient prognosis [36]. IL-1β in the tumor microenvironment potentiates carcinogenesis by promoting local inflammatory responses and stimulating angiogenesis. In addition, a study showed that co-culture of macrophages with breast cancer cell lines enhances IL-1β expression, IL-1β-dependent induction of cyclooxygenase (COX)-2 and tumor progression [37]. Although macrophages are known to be the major cells secreting IL-1β in tumor microenvironment, tumor cells such as prostate, breast, and head & neck cancer cells are also able to produce IL-1β to potentiate their progression and invasiveness [38]. We observed sustained IL-1β expression by THP-1, but not NPC cells (Figures 5B and S4A), suggesting that different types of cancer cells have different ability to produce this factor. However, NPC-derived factors are able to induce IL-1β expression in monocytes/macrophages and direct interaction between monocytes/macrophages and NPC cells greatly increased the expression of IL-1β, indicating that NPC cells are able to “educate” macrophages by both secretory factors and cell-cell contact to induce IL-1β. In line with increased expression and secretion of IL-1β, we observed increased expression of AIM2 and Caspase-1 genes in monocytes/macrophage-NPC co-culture. AIM2 protein is part of the inflammasome, a multiprotein oligomer that is activated by microbial infection or detection of danger signals such as extracellular ATP or reactive oxygen species (ROS) [39]. Activation of AIM2 and the subsequent assembly of the inflammasome complex activate caspase-1 for the release of IL-1β. In our study, the secretion of IL-1β occurs prior to the induced increase of AIM2 and CASP1 expression (Figure 1B, 2C, 3A). It is possible that other pathways are responsible for the early secretion of IL-1β and further induction of IL-1β by the AIM2 pathway helps to maintain or exacerbate the chronic inflammation seen in NPC [36].

High levels of MCP-1 has been associated with poor prognosis in various cancers such as breast, colon, prostate and cervical cancer [40]. MCP-1 regulates cancer progression and metastasis through various mechanisms including recruitment of inflammatory cells, immune suppression, angiogenesis and metastasis [40]. For instance, direct induction of VEGF-mediated angiogenesis has been proposed to be dependent on MCP-1 production and macrophage recruitment [41]. Moreover, MCP-1/CCL2-dependent recruitment of monocytic cells has been shown to be important for tumor cell colonization of distant organs in numerous cancers including lungs, bones and liver, although the exact function of the recruited monocytic cells in metastasis is not clear [40]. In NPC, expression of MCP-1 was reported to be found predominantly in infiltrated macrophages [32], which coincide with our data where we observed that MCP-1 was predominantly expressed in THP-1 during macrophage-NPC interaction (Figures 5B and S4A). Both contact and transwell co-cultures of THP-1 and C666-1 cells induced high expression of MCP-1 (Figures 3A, 5B, S4A), indicating that soluble factors from NPC cells are sufficient for induction of MCP-1 expression in macrophages to sustain monocyte recruitment. Interestingly, at 96 hrs after co-culture, MCP-1 mRNA expression was observed in C666-1 cells as well (Figure S4A), suggesting that long-term interaction between macrophages and NPC cells is able to induce MCP-1 expression in NPC cells. These results imply that there is a prolonged signal for the recruitment of circulating monocytes into the NPC tumor tissue where the monocytes subsequently differentiate into macrophages (TAMs) to exert its effects in tumors.

Inflammation stimulates monocytes/macrophages to express IL-10, a potent anti-inflammatory cytokine which plays a major role in preventing uncontrolled inflammation [42, 43]. IL-10 inhibits the expression of inflammatory cytokines and chemokines including IL-1β, TNFα and MCP-1, suppresses antigen presentation and CD4^+^ T cell activation and function. On the other hand, IL-10 enhances proliferation and cytotoxic function in CD8^+^ T cells [44, 45]. Interestingly, we only observed transiently induction of IL-10 at 1 and 3 hrs after THP-1-C666-1 cell interaction (Figure 1B, 3A, S3A). The early (3 hrs) induction of IL-10 during primary monocyte-NPC cell interaction was also observed, although it was peaked at 24 hrs post-co-culture and was still higher than basal levels at 48 hrs (Figure 4A). These results suggest that NPC cells are able to suppress IL-10 expression to sustain local inflammation and possibly to suppress cytotoxic T cell activity. The mechanism on the downregulation of monocyte IL-10 expression by NPC cells requires further investigation. TGFβ is another important immunosuppressive cytokine produced by TAMs in various tumors [46, 47]. Interestingly, we do not observe induction of TGFβ in our macrophage-NPC cell co-cultures (Figure S2). Together, our study reveals that monocyte/macrophage-NPC cell interaction favors the development of sustained inflammation. Such inflammation may be important for progression and metastasis of NPC.

In addition to cancer-promoting inflammatory mediators, TAMs secrete MMPs including MMP9 to promote metastasis [34]. We observed increased and sustained expression of MMP9 upon interaction between macrophages and NPC cells in our co-culture systems. MMP9 belongs to the MMP family of zinc-dependent endopeptidases that digests the extracellular matrix and has been associated with tumor cell invasion, metastasis, and tumor-induced angiogenesis [48]. Increased expression of MMP9 has been detected in invasive/metastatic cancers such as colorectal cancer [49], gastric carcinoma [50], pancreatic carcinoma [51–53], breast cancer [51], and oral cancer [54]. It has been reported that MMP9 can be induced by the interaction between macrophages and cancer cells. For instance, co-culture of macrophages with ovarian or breast cancer cell lines was found to increase the expression of MMP2 and MMP9 in macrophages, but not in tumor cells, to promote tumor invasiveness [55]. In breast cancer, tumor cell-derived MMP9 drives malignant progression and metastasis of basal-like triple negative breast cancer [56]. In NPC, MMP9 is associated with NPC progression [57, 58], nodal metastasis [59, 60] and increased cell invasion *in vitro* [61]. Together, these studies demonstrate that MMP9 can be produced by both TAMs and tumor cells for cancer progression and metastasis. In this study, we observed MMP9 expression in both THP-1 and C666-1 cells but with different expression kinetics. In THP-1 cells, it was induced at 6 hrs upon their interaction with NPC cells and its expression was continuously increased (Figures 5C and S4B). The induction of MMP9 expression in NPC cells appears to be much later. We observed significant induction of this gene in NPC cells after 48 hrs of contact co-culture (Figure 5C), whereas its induction was only observed after 96 hrs of non-contact co-culture (Figure S4B). These results suggest differential regulation of MMP9 expression in TAMs versus tumor cells as well as between contact and non-contact co-culture systems.

Importantly, we observed that several other cancer-promoting genes including CTGF, PLAU and PLA2G7 were induced in macrophages during macrophage-NPC interaction (Figures 2–5). CTGF is a key component of wound repair and has been implicated in tumorigenesis [62, 63]. PLAU encodes the urokinase-type plasminogen activator (uPA) which leads to proteolytic degradation of the extracellular matrix (ECM) [64, 65]. uPA converts the proenzyme plasminogen into the serine protease plasmin, which functions by degrading fibrin and activating MMPs, and thus has been implicated in tumor invasion and metastasis [64–66]. Moreover, uPA and MMP9 expression by TAMs in breast and lung cancers correlated with increased angiogenesis and invasion, and poor patient survival [67–70]. However, CTGF and PLAU expression were observed to be transiently upregulated during the early phases of co-culture (Figures 2C, 3B, 5C). PLA2G7, on the other hand, was found to be induced at 24 hrs post co-culture and its expression was sustained thereafter (Figures 2–5) The majority of these cancer-promoting factors were also found to be expressed mainly in the THP-1 cells after co-culture with the NPC cells (Figures 5C, S4B, S5B).

We next examined the ability of the soluble factors produced by macrophages to enhance cell migration. Previous studies have shown that conditioned media from M2-polarized THP-1 cells/human macrophages or unpolarized macrophages induced increased cell migration and invasion in various cancer cell lines including colon [71, 72], breast [73] and pancreatic cancers [74]. In contrast, M1 conditioned media from THP-1 led to growth inhibition and apoptosis in colon cancer cells [75]. In this study, we found that the migration of C666-1 cells was enhanced by conditioned media from THP-1 compared to cells incubated with complete media (Figure 6A). This suggests that soluble factors produced by the macrophages at steady state are able to induce increased migration capabilities of the cancer cells. Furthermore, conditioned medium from the co-cultures led to further enhancement in NPC cell migration (Figure 6A and 6B). This suggests that the increased production of factors from the monocytes/macrophages due to monocyte/macrophage-NPC cell interaction promotes cancer cell migration in NPC which may contribute to increased tumor aggressiveness.

The PLA2G7 gene encodes a phospholipase A2 enzyme, also known as platelet-activating factor acetylhydrolase (PAF-AH), which hydrolyzes and converts PAF to lyso-PAF [76]. In prostate cancer, PLA2G7 is highly enriched in primary tumor samples, but not in the adjacent normal tissues, and is positively associated with poor survival and more aggressive disease [27]. In colorectal cancer (CRC), increased activity and expression of PLA2G7 were detected in tumor tissues and plasma from CRC patients compared with samples from healthy donors. In addition, absence of PLA2G7 in APC^min/+^ mice resulted in reduced intestinal tumorigenesis [28], providing further evidence of tumor-promoting function of PLA2G7 in CRC. However, the source of this enzyme has not been identified. In addition, the function of PLA2G7 in cancers other than colorectal cancers has not been reported. Our results demonstrate that THP-1-C666-1 interaction leads to induction of the PLA2G7 gene and increased secretion of PLA2G7 protein from monocytes/macrophages (Figures 5C, S4B, S5B, 6C).

Following this, the ability of the PLA2G7 to enhance cell migration was examined. Conditioned media from THP-1 cells, supplemented with the concentration of PLA2G7 derived from our ELISA assay, induced greater C666-1 cell migration and would healing than conditioned media from THP-1 cells alone (Figure 6D–6E), suggesting that PLA2G7 may enhance NPC cell migration together with other factors produced by monocytes/macrophages. Similarly, HK-1 cells incubated with conditioned media from THP-1 cells supplemented with PLA2G7 displayed greater cell migration than when incubated with conditioned media from THP-1 cells alone (Figure S7). Interestingly, enhanced C666-1 migration was only induced by THP1 conditioned medium supplemented with 80 pg/mL PLA2G7 but not normal culture medium with 80 pg/mL PLA2G7 (Figure S6). This suggests that PLA2G7 might work in conjunction with other factors present in the THP1 leading to enhanced NPC cell migration. In agreement with our findings, the migration of C666-1 cells was reduced when incubated with conditioned media from *PLA2G7*-silenced THP-1 cells compared with conditioned media from control transfected THP-1 cells. A reduction in C666-1 migration was also observed when incubated with conditioned media from co-culture of *PLA2G7*-silenced THP-1 cells with C666-1 as compared to conditioned media from control-transfected THP-1 cells co-cultured with C666-1, which confirms our findings that PLA2G7 plays an important role in regulating cell migration in NPC (Figure 7A and 7B). The conditioned media from control transfected and PLA2G7-silenced THP-1 cells co-cultured with C666-1 cells induced greater cell migration than conditioned media from control-transfected and PLA2G7-silenced THP-1 cells respectively, indicating that other factors produced by the monocyte/macrophages play a role in regulating NPC cell migration (Figure 7A and 7B). The increase in number of migrated C666-1 cells into the tissue culture wells was not due to increased proliferation of the cells as evidenced in the crystal violet staining (Figure S8). In agreement with our study on the role of PLA2G7, Vainio *et al.* also previously showed that PLA2G7 silencing reduced cell migration and motility in prostate cancer cells [27]. Additionally, we found that gene silencing of PLA2G7 in THP-1 cells led to reduced induction of inflammatory cytokine genes after co-culture with C666-1 cells (Figure 7C). Our results demonstrate that NPC cells induce the expression of PLA2G7 in monocytes/macrophages during monocyte/macrophage-NPC interaction to promote migration of NPC cells, which might contribute to tumor invasiveness or metastasis.

Interestingly, we found that breast cancer cell MCF and non-small lung cancer cell H1299 were also able to induce THP-1 cells to express PLA2G7 (Figure 7D). Therefore, the induction of PLA2G7 expression in TAMs by cancer cells could be an important factor in tumor-promoting function of TAMs in various cancers.

In summary, we show that the interaction between macrophages and NPC cells leads to induction of various inflammatory and cancer-related genes in macrophages, suggesting that monocytes/macrophages that are recruited into the NPC tumor microenvironment are “educated” to become tumor promoting cells. Importantly, we identified macrophage-derived PLA2G7 as a factor that promotes NPC cell migration, suggesting that PLA2G7 may provide a novel therapeutic target in the treatment of NPC. In addition, we found that the expression of TAM-associated immunosuppressive cytokines including IL-10 and TGFb was not induced or not sustained during macrophage-NPC interaction. Furthermore, the M2 macrophage-associated enzyme, arginase 1, was not induced during macrophage-NPC interaction. Furthermore, we found that CRC cells HCT116 and SW480 who are constitutively express PLA2G7 do not induce THP-1 cells to expression this gene, whereas breast cancer cell MCF7 and non-small lung cancer cell H1299 who do not expression PLA2G7 themselves are able to induce this gene in THP-1 cells. These results, together with previous reports on the diverse phenotypes of TAMs in various types of cancers [8, 10, 77], suggest that the specificity of TAMs in a given cancer should be taken into consideration when targeting TAMs for the development of anti-cancer therapy.

## MATERIALS AND METHODS

### Cell culture

THP-1 monocytic cells were purchased from ATCC. C666-1, an EBV positive undifferentiated nasopharyngeal cancer cell line was obtained from Professor Chan Soh Ha (NUS Immunology Centre). All cell lines were cultured in RPMI 1640 (Invitrogen, Carlsbad, CA) supplemented with 10% FCS, 100 mg/ml penicillin/streptomycin (complete medium).

Human PBMCs were isolated from normal blood donor buffy coats using Ficoll density gradient (GE Healthcare). Monocytes (~98% purity) were isolated using anti-CD14 microbeads (Miltenyi Biotec, Bergisch Gladbach, Germany).

To isolate THP-1 cells from C666-1 cells in our contact- co-cultures, THP-1 cells were labeled with 2.5 μmol/L CFSE (Molecular Probes, Eugene, OR) for 10 minutes at 25°C in a 0.1% bovine serum albumin (BSA)/phosphate-buffered saline (PBS) buffer before co-culture. The CFSE+ (THP-1) and CFSE- (C666-1) fractions were isolated and analysed by flow cytometric sorting (Beckman Coulter).

To generate THP-1 macrophages, THP-1 cells were treated with 100 ng/mL phorbol 12-myristate 13-acetate (PMA; Sigma) for 48 hours. The cells were then washed 3 times with serum-free media to remove all traces of PMA and cultured in complete RPMI for another 48 hours before use in subsequent experiments.

### Monocyte/macrophage cells co-culture with NPC cell lines

THP-1 or primary human monocytes were cocultured with NPC cell lines to mimic an environment of NPC tumor infiltrated with monocytes/macrophages. For direct contact coculture, the NPC cells were cultured together with THP-1 cells or human monocytes at a 1:1 ratio in a 6-well plate. For the 0hr coculture time point, THP-1 and C666-1 were cultured and harvested separately. The harvested cell pellets were subsequently pooled together for RNA extraction. For non-contact coculture, 0.2 × 10^6^ C666-1 were seeded in the lower chamber and 0.2 × 10^6^ THP-1 cells or human monocytes were seeded in the upper inserts of a 6-well Transwell plate (Corning) followed by culturing over the required time. For culture of THP-1 cells in C666-1 conditioned media, 0.2 × 10^6^ C666-1 cells were cultured for every 2 ml complete RPMI medium for three days. The medium was harvested and filtered through 0.22 μm filter for use as C666-1 conditioned medium.

### Real-time polymerase chain reaction

Real-time polymerase chain reaction (qPCR) was used to examine the expression of genes associated with inflammation and cancer progression. Total RNA was isolated from the cells using TRIzol reagent (Invitrogen) according to manufacturer's instructions. 1 μg of RNA was reverse transcribed into complementary DNA (cDNA) to be used for qPCR. qPCR was performed using SsoAdvanced SYBR Green Supermix and CFX Connect RT System (BioRad). The following primers were used for qPCR: Actin, Forward primer (FP): CCCCGCGAGCACAGAG, Reverse primer: ATCATCCATGGTGGTGAGCTGGC; AIM2, FP: CTGCAGTGATGAAGACCATTC, RP: CA TTTCTGATGGCTGCAGATG; CASPASE-1, FP: CAGC ACGTTCCTGGTGTTC, RP: GATGATCACCTTCGGTT TGTC; CD44, FP: CAACCACAAGGATGACTGATG, RP: CTTCTGGGTAGCTGTTTCTTC; CTGF, FP: CCAAC TATGATTAGAGCCAACTG, RP: GTTCTCTTCCAGGT CAGCTTC; IL-1β, FP: GTGGCAATGAGGATGACTTG, RP: GCAGGGAACCAGCATCTTC; IL-6, FP: AGGAG ACTTGCCTGGTGAAA, RP: CAGGGGTGGTTATTG CATCT; IL-10, FP: GCTCAGCACTGCTCTGTTG, RP: CACTCTGCTGAAGGCATCTC; MCP-1, FP: ATGAAA GTCTCTGCCGCCCTTC, RP: CTGCTTGGGGTCAGC ACAGATC; MMP9, FP: CAACTACGACCGGGACAAG, RP: CTTCTTGTCGCTGTCAAAGTTC; PLAU, FP: CCA AAGAAGGAGGACTACATC, RP: GATCTTCAGCAAG GCAATGTC; PLA2G7, FP: GTCTTGGCTCTACCTTA GAAC, RP: CTTCACTGGCTTTCCATGATC; TNFα, FP: TCCTTCAGACACCCTCAACC, RP: AGGCCCCAGTTT GAATTCTT; VEGFα, FP: CAGCTACTGCCATCCAAT CG, RP: GCATAATCTGCATGGTGATGTTG. Actin was used as the internal control.

### Microarray gene expression profiling

RNA isolated from the THP-1/NPC C666-1 cell cocultures were used to conduct microarray gene expression profiling using the Illumina BeadArray (Human HT-12v4)

### Determination of protein levels in cell culture medium by ELISA

The levels of IL-1β and PLA2G7 secreted into the culture supernatant were measured using enzyme-linked immunosorbent assay kit from R&D Systems (Minneapolis, Minnesota, USA).

### Gene knock-down using RNA interference

Specific gene knock-down of PLA2G7 was performed using siRNA molecules (SI00072177, AAGGACTCTATTGATAGGGAA) (Qiagen GmbH, Hilden, Germany). AllStars Negative Control scrambled siRNA (Qiagen) was used as a negative control. For cell transfection, 0.2 × 10^6^ THP-1 cells were seeded into each well of a 12-wellplate. The cells were incubated with the siRNAs and Lipofectamine 3000 Transfection Reagent (Invitrogen, Madison, WI, United States). Transfected cells were grown at 37°C overnight, followed by incubation with complete medium for 24 h before experiments.

### Transwell migration and cell proliferation assay

Serum starved C666-1 cells were added into transwell in serum free RPMI and allowed to migrate through to the culture wells containing either complete medium (control), conditioned medium from cultured cells or medium supplemented with PLA2G7 for five days. Migrated C666-1 cells were photographed under an inverted microscope (20× objective, Olympus) and quantitative assessment of cell number were determined by standard MTT assay.

For determination of cellular proliferation, C666-1 cells were seeded and serum starved for 24 hr (Day 0) before complete medium, or medium supplemented with PLA2G7 was added to the cells. At the end of each time point, the media was aspirated and the cells washed once with PBS. The cells were then stained with 1% crystal violet (Sigma) in 20% methanol for 5 minutes before the excess stain was washed off with water and air-dried. The retained crystal violet was solubilized using 0.1 M sodium citrate in 50% ethanol. Absorbance readings were taken at 535 nm to measure the amount of crystal violet taken up by the viable cells. The results were normalized to cells that were stained at 0 h after serum starvation.

### Wound healing assay

Uniform wounds were introduced into the cell culture system by using the ibidi Culture-Insert (ibidi GmbH Munich, Germany, No. 80209). Each cell culture insert provides two cell culture reservoirs with a separation wall of 500 μm thick. One insert is placed in each individual well of a 24-well plate. 4000 C666-1 cells were cultured with a volume of 70 μL medium in each reservoir. The silicon inserts were removed after the cells have adhered to the plate. The resulting gaps (or wounds) were washed with serum-free medium and each well was filled with 1 mL of culture medium, or conditioned medium from THP-1 cells, NPCs cells or co-cultures of THP-1 and NPC cells. At different time-points, the cells that have migrated into the gap were photographed (10× objective) using a Meta Morph controlled Nikon camera (Fluorescence Inverted microscope (Nikon 2000 E, Nikon GmbH Duesseldorf, Germany). The closing areas were calculated using ImageJ software (http://rsbweb.nih.gov/ij/) and the data was incorporated into Excel program for further analysis.

### Statistical analysis

The results are presented as the mean ± SD. Statistical analysis was performed using Kruskal Wallis non-parametric analysis with Dunn's multiple comparisons test.

## SUPPLEMENTARY MATERIALS




