# Supplementary material for: Monocyte-derived factors including PLA2G7 induced by macrophage-nasopharyngeal carcinoma cell interaction promote tumor cell invasiveness
Source: Oncotarget. 2016 Aug 1;7(34):55473–90. doi: 10.18632/oncotarget.10980 (PMC5342430; doi:10.18632/oncotarget.10980)
Supplement: Supplementary file 1 [file oncotarget-07-55473-s001.pdf]

Monocyte-derived factors including PLA2G7 induced by macrophage-nasopharyngeal carcinoma cell interaction promote tumor cell invasiveness

Supplementary Materials

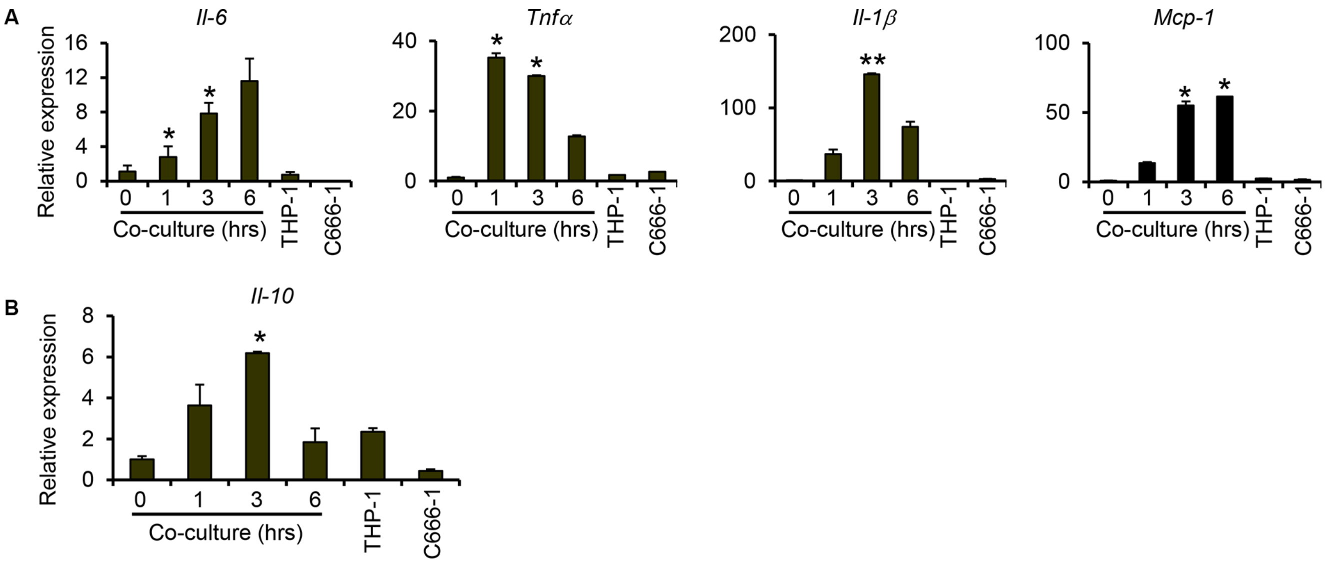

Supplementary Figure S1: Co-culture of C666-1 cells with PMA-differentiated THP-1 cells increases proinflammatory cytokine expression. THP-1 monocytes were differentiated into macrophages with PMA (as detailed in Materials and Methods). The macrophages were then co-cultured with C666-1 NPC cells. Relative gene expression levels of proinflammatory cytokines were determined by quantitative real-time PCR (qPCR). The results shown are a representative set from 2 experiments $*P < 0.05$, $**P < 0.01$ (mean ± S.D, $n = 3$).

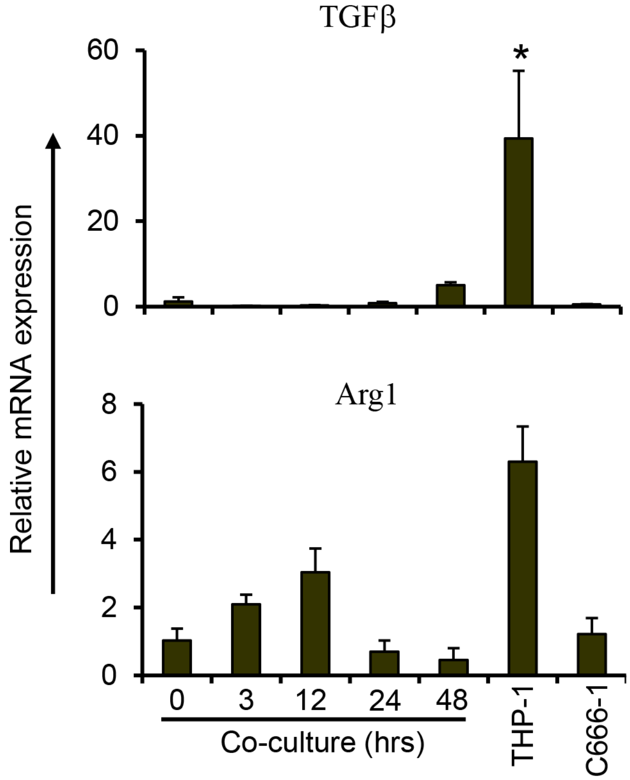

Supplementary Figure S2: Co-culture of C666-1 cells with undifferentiated THP-1 cells leads to increased expression of *Arg1* but not *TGFβ*. Undifferentiated THP-1 cells were co-cultured with C666-1 NPC cells and relative gene expression levels of *Arg1* and *TGFβ* were determined by quantitative real-time PCR (qPCR). The results shown are a representative set from 2 experiments *$P < 0.05$ (mean ± S.D, $n = 3$).

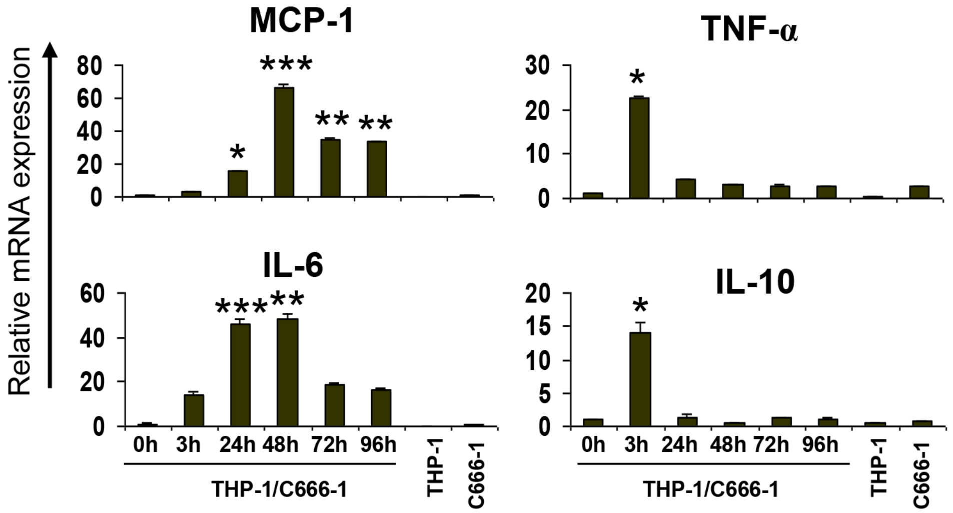

Supplementary Figure S3: Prolonged co-culture of undifferentiated THP-1 cells with C666-1 cells increased expression levels of proinflammatory cytokines and the chemokine, MCP-1. Undifferentiated THP-1 cells were co-cultured with C666-1 cells over 4 days. Expression of *MCP-1, IL-6, TNFa* and *IL-10* genes was assessed by qPCR. The qPCR data shown is a representative set of 2 experiments (mean ± S.D, $n = 3$) *$P < 0.05$, **$P < 0.01$, ***$P < 0.001$ (mean ± S.D, $n = 3$).

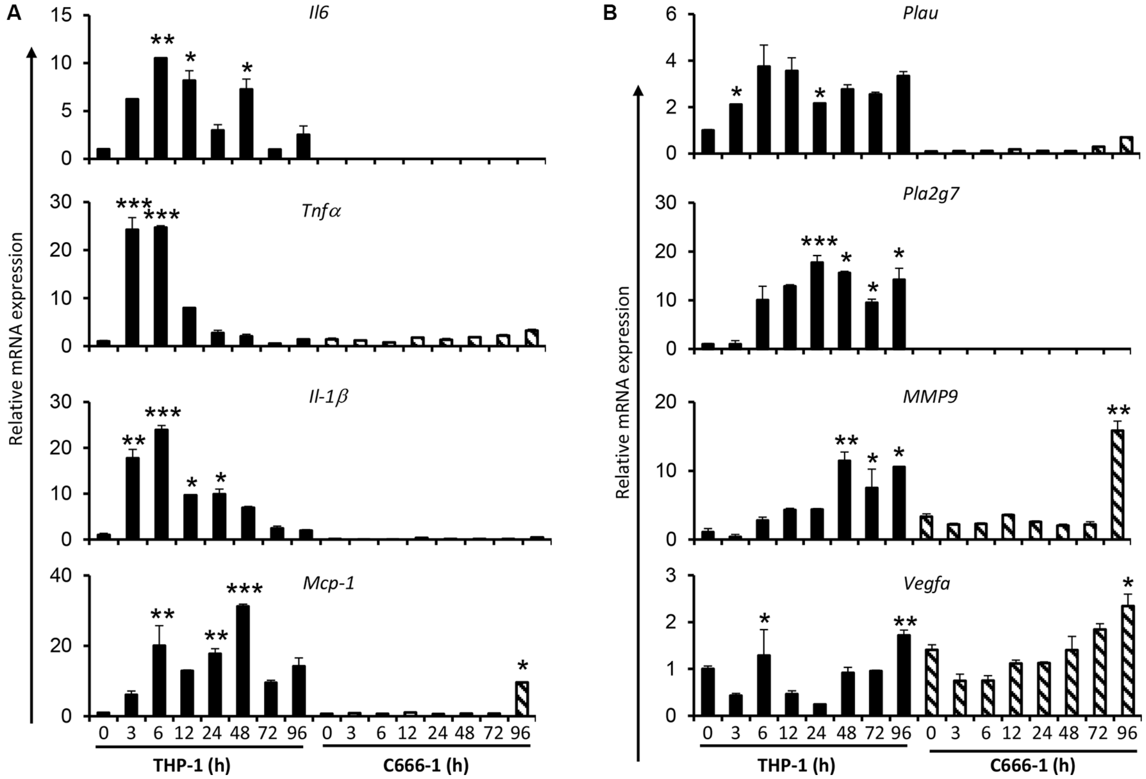

Supplementary Figure S4: Gene expression by THP-1 cells or NPC cell during non-contact co-culture. Non-contact co-culture of undifferentiated THP-1 and C666-1 cells was conducted in a transwell system. Expression levels of (**A**) inflammatory, or (**B**) cancer-promoting genes by THP-1 cells or C666-1 cells were determined by qPCR. The data shown is representative from a set of 2 experiments (mean ± S.D, $n = 3$) *$P < 0.05$, **$P < 0.01$, ***$P < 0.001$ (mean ± S.D, $n = 3$).

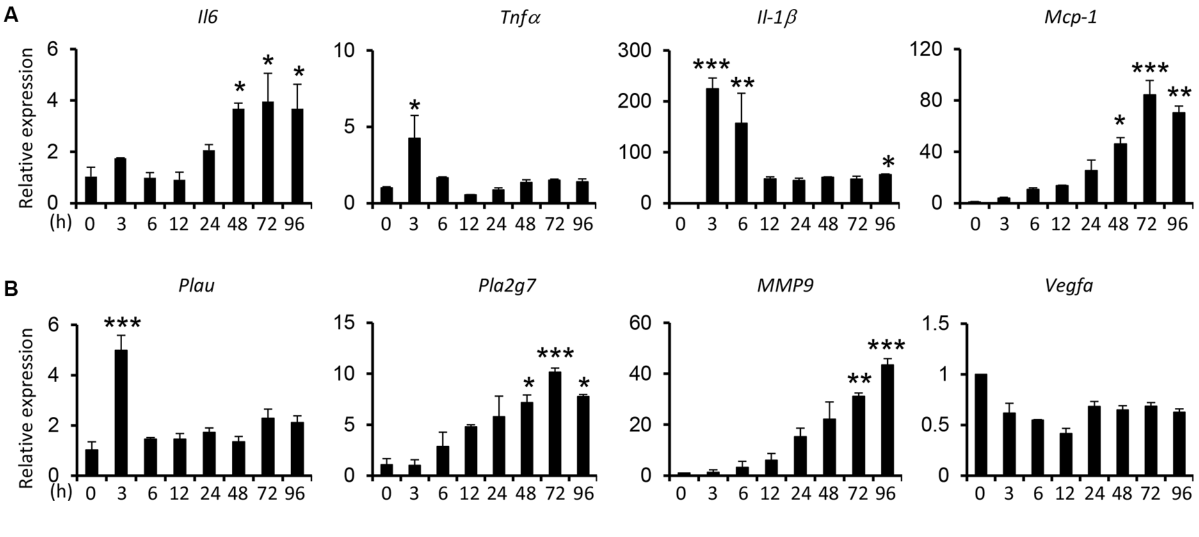

Supplementary Figure S5: NPC-conditioned medium induced the expression of proinflammatory and cancer-promoting genes in THP-1 cells. THP-1 cells were cultured in complete medium or C666-1-conditioned medium over the indicated time periods. Expression of (**A**) pro-inflammatory or (**B**) cancer-promoting genes was determined by qPCR. The results are a representative set from 2 experiments (mean ± S.D, $n = 3$) *$P < 0.05$, **$P < 0.01$, ***$P < 0.001$ (mean ± S.D, $n = 3$).

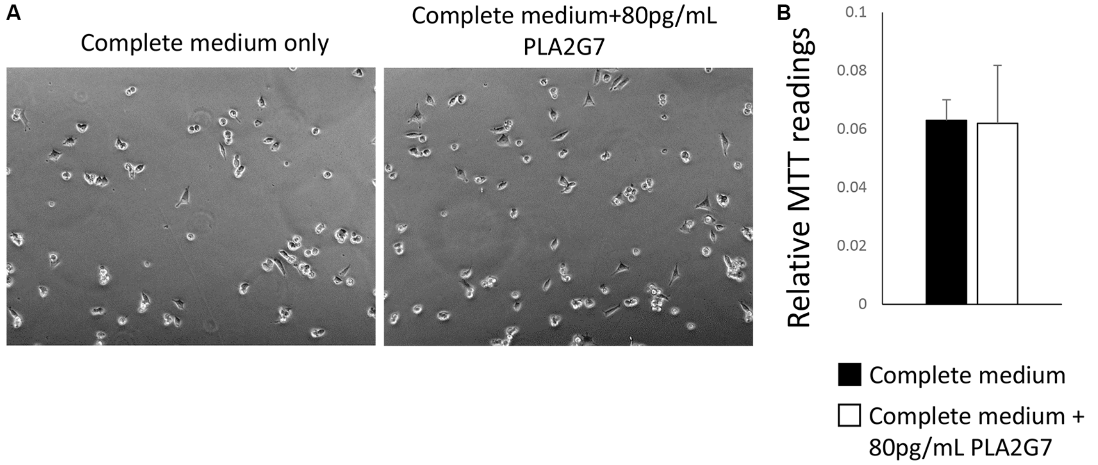

Supplementary Figure S6: PLA2G7 alone was not able to enhance NPC cell migration (A) C666-1 cells were seeded into the transwell (upper insert). Complete medium containing recombinant PLA2G7 was added to the lower wells of the plate and migration of the C666-1 cells across the transwell was determined. (**B**) The representative micrographs and the bar chart show migrated C666-1 cells and cell counts into the lower chamber. Images shown are representative of a set of 3 images from 2 experiments $P > 0.05$ (mean ± S.D, $n = 3$).

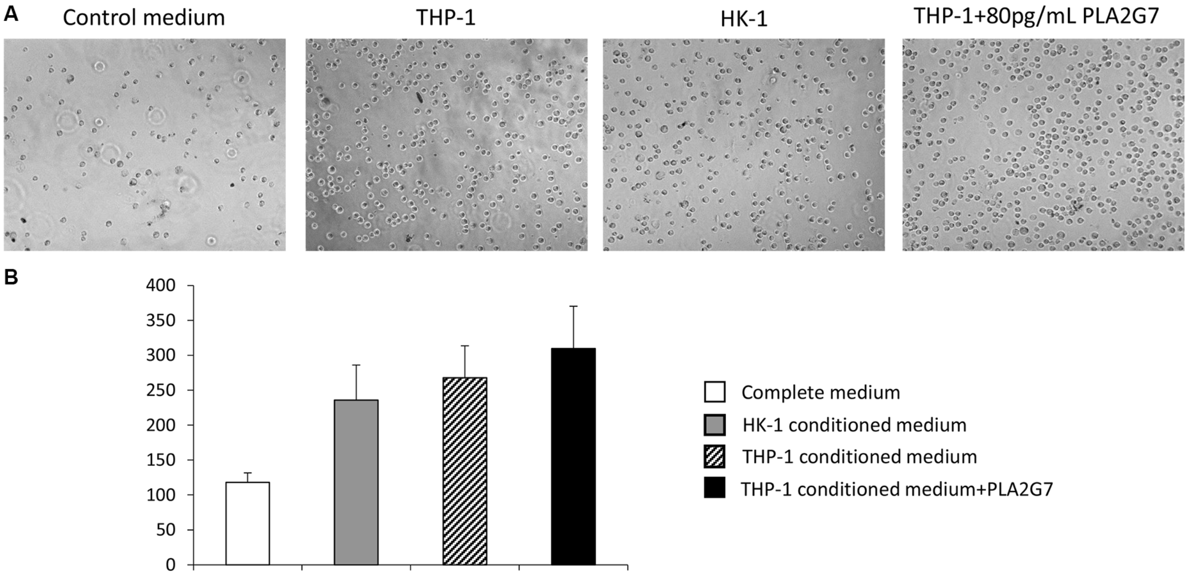

Supplementary Figure S7: Culture medium from THP-1 supplemented with PLA2G7 enhances NPC cell migration. (**A**) HK-1 cells were seeded into the transwell (upper insert). The indicated conditioned medium was added to the lower wells of the plate and migration of the HK-1 cells across the transwell was assessed. (**B**) Representative micrographs and the bar chart show migrated HK-1 cells and cell counts in the lower chamber (migrated cells) relative to the control (complete RPMI medium only) respectively. Images shown are representative of 2 experiments. $*P < 0.05$ (mean ± S.D, $n = 3$).

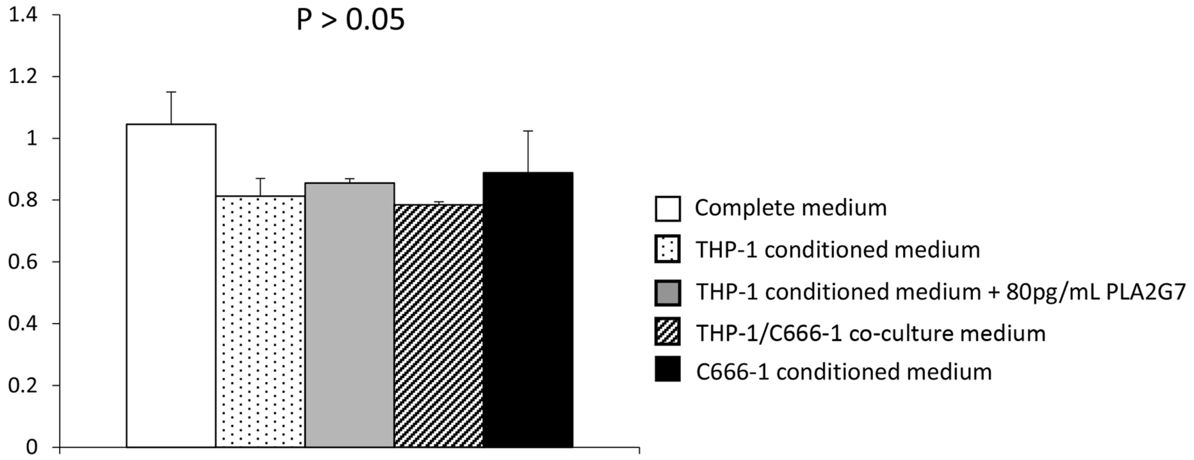

Supplementary Figure S8: Culture of C666-1 cells with recombinant PLA2G7 did not induce significant changes in cell proliferation. C666-1 cells were serum starved for 24 hr before culture in the respective media. At the end of each time point, the cells were stained with 1% crystal violet and 0.1 M sodium citrate in 50% ethanol was used to solubilize the crystal violet stain retained by the cells. Absorbance readings were taken at 535 nm. The results were normalized to cells that were stained at 0 h after serum starvation. The difference in cell proliferation between C666-1 cells cultured in complete medium and in medium supplemented with recombinant PLA2G7 was not significant. The data is representative of 2 experiments. $P > 0.05$ (mean ± S.D, $n = 4$).

Supplementary Table S1 : Microarray analysis of inflammatory and cancer-related genes from 3 hr- and 48 hr-co-culture samples compared to controls (0 hr co-culture). See Supplementary Table S1
